# Advances in the Experimentation and Numerical Modeling of Material Joining Processes

**DOI:** 10.3390/ma17010130

**Published:** 2023-12-27

**Authors:** Raul D. S. G. Campilho

**Affiliations:** 1 ISEP—School of Engineering, Polytechnic of Porto, R. Dr. António Bernardino de Almeida, 431, 4200-072 Porto, Portugal; rds@isep.ipp.pt; Tel.: +351-939-526-892; 2 INEGI—Institute of Science and Innovation in Mechanical and Industrial Engineering, Pólo FEUP, Rua Dr. Roberto Frias, 400, 4200-465 Porto, Portugal

Material joining processes are a critical factor in engineering structures since they influence such structures’ structural integrity, performance, and longevity [1]. While significant progress has been made in the improvement of diverse joining processes [2], current design limitations require further consideration and innovative solutions [3]. This Special Issue, entitled “Advances in the Experimentation and Numerical Modeling of Material Joining Processes”, published by MDPI, aims to explore the current state of these processes, identify design limitations, propose avenues for improvement, and discuss ongoing research and prospects. Both experimental and numerical simulation approaches are examined to provide a complete perspective of the field. This Special Issue addresses a wide scope of joining methods, ranging from traditional techniques such as welding and brazing to modern innovations such as friction stir welding (FSW) and laser welding. Historically, mechanical fastening and welding techniques have been indispensable in material joining. Mechanical fastening stands out as a prevalent approach in the industry [4]. Initially employed for metal joining, this method has gained acceptance for use in the production of composite materials [5]. Screws are a robust and cost-effective non-permanent solution, although they result in negative effects with regard to weight and aesthetics [6]. Permanent fastening mechanisms, such as snap-fits, are incorporated directly into components during molding, increasing durability and eliminating the risk of loosening over time [7]. Variants of traditional welding include extrusion welding and gas–metal–arc welding, which are in prevalent use [8,9]. FSW, a solid-state joining process, excels in joining difficult-to-weld materials through use of traditional methods [10]. Laser welding presents a high-energy-density approach, allowing for precise control and minimal thermal distortion [11]. Hybrid joining techniques, e.g., combining laser and arc welding, are gaining prominence [12]. Adhesive bonding has gained increasing importance in engineering, with it offering weight reduction and improved fatigue performance [13]. Associated with all joining processes, the integration of numerical modeling is crucial for a deeper understanding of complex phenomena. In fact, computational simulations help to predict strength and analyze thermal distributions, stress concentrations, and material flow during joining processes [14,15].

Current design limitations

- Traditional screws and fasteners add weight and may pose size constraints when lightweight and compact designs are crucial, such as in the aerospace and electronics industries [16]. In some cases, mechanical fasteners may be sensitive to temperature variations, leading to thermal expansion or contraction, which can affect joint integrity [17].
- Welding and brazing often face challenges related to heat-affected zones (HAZs), residual stresses, and microstructural alterations [18]. Despite extensive industrial application, strength and material compatibility limitations exist [19].
- FSW has benefits such as reduced heat input and minimized distortion [20] but is limited by tool wear, process stability, and adaptability to different materials, which pose significant design challenges [21].
- Laser welding, as a high-energy-density approach, presents a unique set of design limitations, such as optimal parameter selection to balance efficiency and thermal stress [22]. Hybrid laser welding introduces additional complexities, namely the interactions between different heat sources, equipment synchronization and coordination, and welded material compatibility with different processes [23].
- Adhesive bonding faces challenges related to joint strength, durability, loading rate, and process optimization [24], added to the non-existing standardized adhesive joint characterization protocols. The comparability of results becomes difficult due to variations in testing methods and reporting parameters [25]. Furthermore, the absence of a standardized predictive approach presents challenges in the design process [26].
- In the numerical modeling domain, challenges persist in accurately representing the material joining processes, due to recurring simplifying assumptions that limit the predictive capabilities [27]. Computational simulations require refinement and need to account for all associated phenomena to enhance predictive accuracy [28].

Improving existing design processes

- In mechanical fastening, increasing durability and fatigue strength are essential for applications under repetitive loading or dynamic stress [29]. Industrially, simplifying the assembly process by designing fastening mechanisms that are user-friendly and require minimal manual effort is a crucial aspect to enhance productivity [30].
- Welding and brazing can benefit from studies on the optimization of process parameters, including heat input and post-weld cooling rates, with the aim of mitigating challenges associated with HAZ and respective microstructural alterations, and residual stresses in the welded parts [31]. Integrating advanced materials and alloys can increase joint strength and material compatibility, leading to robust and reliable designs [32].
- In FSW, tool wear and process stability issues are crucial for process improvement [33]. Research efforts are being made to develop wear-resistant tool materials and optimize tool geometries [34]. An improved understanding of material flow during FSW may improve control over the process, ensuring high-quality joints [35].
- Laser welding process improvement involves balancing the process parameters to achieve optimal efficiency and minimal thermal stress [36]. Under this scope, recent advancements in laser technology and real-time monitoring systems have contributed to efficient joining [37]. Hybrid joining between laser welding and other techniques requires understanding process interactions and the exploitation of synergistic effects [38].
- Adhesive bonding can benefit from adhesives with improved strength, durability, and tailoring at the molecular level, offering given requirements for specific applications [39]. Comprehensive design guidelines should be developed [40] together with robust methodologies for adhesive joint characterization, either experimental or numerical [13,41]. Advances in non-destructive evaluation techniques can provide real-time monitoring of joint behavior under operation [42].
- Numerical modeling plays a fundamental role in improving design processes by replicating complex phenomena and reducing the testing requirements [43,44]. Refining computational models and removing any geometrical and material simplifying to accurately simulate material behavior, thermal distributions, and stress concentrations has led to improvements in predictive capabilities [45].

Current lines of research

- In mechanical fastening, recent investigations have used titanium alloys or high-strength stainless steels to enhance fastener properties, such as improving strength, reducing weight, and increasing corrosion resistance [46]. Lightweight materials and composites as fasteners are an ongoing topic of research to achieve a high strength-to-weight ratio [47].
- Traditional welding topics include advanced filler materials, gas compositions, and shielding atmospheres, with the aim of enhancing the high robustness and quality of welds [48]. Research works on innovative heat management methods, including pulsed welding and tailored energy inputs, show potential to minimize thermal distortions [49].
- Research on FSW is focused on understanding and mitigating tool wear [50], namely through the proposal of innovative tool geometries and adaptive control strategies, which extend tool life and maintain process stability [51]. New tool materials to improve process behavior under varying process conditions are also addressed [52].
- Laser welding investigations focus on optimizing beam characteristics, such as wavelength and focus, to increase precision and control [53]. Real-time monitoring and feedback systems are being developed to dynamically adjust the process parameters, ensuring consistent and high-quality joints [54]. The integration of robotics into hybrid laser welding systems is gaining attention for use in increasing production rates [55].
- Adhesive bonding research is currently exploring advanced and tailored adhesive formulations with improved mechanical properties, durability, and adaptability to diverse substrates [56]. Novel surface treatments and pre-bonding techniques [57], and adhesive testing procedures under extreme conditions, such as high temperatures or corrosive environments, are other active fields of research [58].
- Computational models are becoming increasingly sophisticated, accurately simulating the complex dynamics of material joining processes [59]. Multiphysics simulations, involving thermal, mechanical, and metallurgical aspects, are innovations in numerical modeling research [60].

Prospects

- Artificial intelligence (AI) and machine learning should bring new insights through integration into numerical modeling to achieve enhanced accuracy and the optimization of the different processes [61]. AI algorithms can learn from vast experimental datasets, enabling the identification of patterns and correlations to improve predictions [62].
- Innovative materials can bring new developments in material joining. The improvement of advanced alloys, composites, and smart materials with tailored properties is ongoing [63], potentially leading to new approaches. Self-healing materials, for example, offer the possibility for joints to self-repair and lifespan extension [64].
- Hybrid joining methods are a promising avenue for the future. The synergistic combination of welding with adhesive bonding [65] or friction stir welding with adhesive bonding [66] presents opportunities to overcome individual method limitations.
- Further investigations of meshless methods and the eXtended Finite Element Method (XFEM) are anticipated to aid in overcoming conventional FEM techniques in evaluation crack initiation and propagation [67]. These methods present alternative approaches to deliver a more precise and efficient representation of joint behavior [68,69].
- Environmental sustainability is a critical aspect in material joining. Efforts are underway toward developing eco-friendly joining processes, which aid in reducing either energy consumption or the environmental impact [70]. Green welding technologies, such as friction stir scribe welding and cold metal transfer welding, aim to reduce the carbon footprint of such processes [71].

In conclusion, this Special Issue, “Advances in the Experimentation and Numerical Modeling of Material Joining Processes”, aims to highlight the state of the art of the most diverse material joining processes, including respective challenges and opportunities. In parallel, this editorial paper has also aided in highlighting the main limitations, suggested improvements to be made, active research lines, and prospects. It has thus been shown that, currently, significant limitations exist but that these limitations also find a response in the current topics being addressed worldwide by academicians and industrialists. Moreover, innovative works are identified that address current topics such as AI, smart materials, hybrid joining, numerical methods, and green technology, which may bring significant improvements in joining processes and help to progress the performance of structures even further.
